# Crania

**Published:** 1860-01

**Authors:** 


*Crania.*—The faculty of the Baltimore College have recently imported from Paris some beautifully prepared crania, which are intended for the purpose of assisting the student in the study of first and second dentition. The collection numbers nearly thirty entire heads, of which a few are still to arrive, ranging from the third month of intra-uterine life to the completion of second dentition. These are prepared in such a manner as to enable one to study the physiological acts relating to the situation and growth of the teeth to great advantage. In this connection we may remark that, while the museum of the college is well supplied with many interesting specimens—anatomical, physiological, and pathological, with numerous maps, plates, drawings, etc.,—acquisitions are made whenever found to be conducive to the wants of instruction.

